# Australian Consumers Are Willing to Pay for the Health Star Rating Front-of-Pack Nutrition Label

**DOI:** 10.3390/nu12123876

**Published:** 2020-12-18

**Authors:** Sheri L. Cooper, Lucy M. Butcher, Simone D. Scagnelli, Johnny Lo, Maria M. Ryan, Amanda Devine, Therese A. O’Sullivan

**Affiliations:** 1 School of Health and Human Sciences, Gold Coast Campus, Southern Cross University, Southern Cross Drive, Bilinga, QLD 4225, Australia; 2 School of Health and Medical Sciences, Edith Cowan University, 270 Joondalup Drive, Joondalup, WA 6027, Australia; lucy.butcher@foodbankwa.org.au (L.M.B.); a.devine@ecu.edu.au (A.D.); t.osullivan@ecu.edu.au (T.A.O.); 3 Foodbank WA, 23 Abbott Road, Perth Airport, WA 6105, Australia; 4 School of Business and Law, Edith Cowan University, 270 Joondalup Drive, Joondalup, WA 6027, Australia; s.scagnelli@ecu.edu.au (S.D.S.); m.ryan@ecu.edu.au (M.M.R.); 5 School of Science, Edith Cowan University, 270 Joondalup Drive, Joondalup, WA 6027, Australia; j.lo@ecu.edu.au

**Keywords:** health star rating, willingness to pay, nutrition labelling, nutrient profiling, consumers, Australia

## Abstract

The Australia and New Zealand Ministerial Forum on Food Regulation has supported the recommendations set out in the 2019 Health Star Rating System Five Year Review Report. Specifically, the forum supported, in principle, Recommendation 9, to mandate the Health Star Rating if clear uptake targets were not achieved while the system is voluntary. Given that mandatory labelling is being considered, it is important to investigate how much consumers value the Health Star Rating in order to understand potential consumer uptake and inform industry. The aim of this study was to assess consumers’ valuation of the Health Star Rating system by analysing their willingness to pay for a packaged food product with the Health Star Rating label, utilising a double-bounded dichotomous choice contingent valuation approach. The results indicate that almost two-thirds of Australian household grocery shoppers were willing to pay more for a product with the Health Star Rating, on average up to an additional 3.7% of the price of the product. However, public health nutrition benefits associated with consumers’ willingness to pay more for products with the Health Star Rating is currently limited by the lack of guarantee of the systems’ accuracy. Given consumer support, a well validated and comprehensive Health Star Rating labelling system can potentially improve health outcomes, cost effectiveness and reduce environmental impacts.

## 1. Introduction

Nutrition labelling is considered a crucial part of the multiple strategies used by policy makers to address diet-related chronic disease [1]. Front-of-pack labels (FoPL) are designed to provide convenient and readily understood nutrition information on packaged foods to encourage healthier food choice and purchase behaviour [2]. There is consistent evidence to suggest that FoPL also influence dietary intakes by providing an incentive for food manufacturers to reformulate products to better align with dietary guidelines [3,4]. Internationally, there are many FoPL in use [5], including the United Kingdom’s Multiple Traffic Light label, where coloured traffic lights are used to categorise the level of individual nutrients in a product [6]; and the Swedish Keyhole label which indicates a product has less salt/fat/sugar and more fibre than comparable products not carrying the symbol [7]. In Australia and New Zealand, the Health Star Rating (HSR) FoPL system aims to provide convenient, relevant and readily understood nutrition information by using a rating from half a star to five stars, where more stars indicate a healthier choice within a given food or beverage category [8,9]. The HSR system is a joint Australian state and territory governments and New Zealand government initiative developed in collaboration with industry, public health and consumer groups [8].

All FoPL schemes are underpinned by one or more nutrient profiling models. Nutrient profiling models are algorithms that are used to classify or rank foods according to their nutritional composition, based on evidence around preventing disease and promoting health [10]. The HSR system is underpinned by six nutrient profiling models which are designed to rank foods specific to their food category [11]. The HSR nutrient profiling models take into account four aspects of a food that are associated with the potential to increase the risk of chronic diseases (energy, saturated fat, sodium and total sugars) along with certain ‘positive’ aspects of a food such as fruit, vegetable, nut and legume content, and in some instances, dietary fibre and protein content [11].

Prior to the implementation of the HSR in Australia in June 2014, the Commonwealth Government appointed a consultancy group to conduct a cost benefit analysis [12]. The purpose of this analysis was to explore the costs to industry, government and non-government organisations, along with the potential benefits for industry and consumers from the implementation of the HSR system. The cost benefit analysis was based on the assumption that HSR implementation would lower health costs by reducing overweight and obesity and other diet-related chronic diseases (e.g., heart disease and type 2 diabetes) [12]. The anticipated benefits from implementing the HSR system were to come from improving consumer nutrition information and food choice behaviour, and improvements to the food supply through product reformulation [12]. The total cost of implementing the HSR system over five years was reported to be 60 million Australian dollars (AUD). Approximately one-third of this cost was allocated to government and non-government organisations who would oversee marketing and promotion, education, enforcement and oversight of the HSR system [12]. The other two-thirds of the implementation costs were attributed to labelling, labour and overheads that would be incurred by manufacturers who voluntarily use the system [12]. Manufacturers may seek to price products to recoup some of these costs. However, prior to the implementation of the HSR, it was unknown if consumers would be willing to pay more for having an HSR on the front of a food package.

In consumer based research, willingness to pay (WTP) estimates indicate the perceived value that a consumer has for a given product or service by providing an estimate of the maximum amount of money they are willing to pay for it [13,14,15]. Mean consumer WTP is an important valuation measure to assess non-marketed resources (i.e., environmental quality, recreation, wildlife, food safety and other food product attributes) and is used to support policy making [13,16,17,18]. In the food science literature, WTP estimates have been used to assess consumers’ value for nutrition and food labelling [19,20,21,22,23,24]. Value elicitation studies, including both direct and indirect methods, are used to determine consumers’ actual or hypothetical WTP [14,15]. There are many ways of measuring WTP, with different approaches having different advantages and limitations [13]. One way of assessing WTP is through a discrete choice experiment (conjoint analysis) which allows the relative importance of individual attributes to be identified, and thereby quantifies consumers’ preferences for commodities [25,26].

The effect of FoPL type (i.e., HSR, Multiple Traffic Light, and Daily Intake Guide) and product healthiness (as represented by the FoPL) on choice and WTP across four foods (cookies, cornflakes, pizza, and yoghurt) was investigated through a discrete choice experiment in previous Australian research [27]. The results showed that of the three FoPL tested, only the HSR resulted in significantly greater WTP for healthier versus less healthy product versions across all foods tested. The results showed that FoPL that are more interpretive, such as the HSR, are more effective at directing consumers towards healthier choices than less interpretive labels. Purely interpretive (or evaluative) FoPL present a recommendation with no nutritional information, whereas purely reductive (non-interpretive) FoPL present reduced nutritional information only (less information than the Nutrition Information Panel) with no recommendation [7]. The authors concluded that the HSR summary indicator, which utilised stars to provide a recommendation, was more effective than the less interpretive Multiple Traffic Lights and the non-interpretive Daily Intake Guide [27]. In a discrete choice experiment, consumers are presented with a sequence of choice sets and are required to choose their preferred alternative in each of the sets presented [26]. Each choice set is defined by a unique combination of attribute levels [25]. For example, in the previous Australian study [27], participants were presented with eight choice sets, each containing images of food packs of the same food type, and were asked to select the item they would like to purchase from the set. Four food types were used in their study (cornflakes, cookies, pizza, yoghurt) and each participant was presented with two choice sets of each food type. Participants were randomised to one of the three FoPL (i.e., Daily Intake Guide, Multiple Traffic Lights or the HSR) and the food pack images contained either the specified FoPL or no label. The food packs also varied according to healthiness (3 levels), health claims (4 levels), price (3 levels) and food type (4 levels). The inclusion of a price attribute allows for a monetary measure of benefit (or WTP) to be estimated from the choices made by the consumers [25].

In Australia and New Zealand, Recommendation 9 of the 2019 HSR System Five Year Review Report states that ‘if the HSR System continues to perform well but the HSR is not displayed on 70% of target products within five years of a government decision of these recommendations, the HSR System should be mandated’ [9] (p. 18). Given that mandatory labelling of the HSR is potentially on the agenda [28], it is important to assess how much consumers value the HSR to understand potential consumer uptake and whether this value differs for different consumers. As such, the primary aim of this study was to assess grocery shoppers’ WTP for a food product with the HSR nutrition label utilising a double-bounded dichotomous choice contingent valuation approach (a type of discrete choice experiment). Although different techniques can be used to measure WTP, contingent valuation is preferred when market data is limited as it uses hypothetical scenarios to elicit people’s preferences for goods/features that are not normally traded [29]. The secondary aim was to examine if there is an association between consumer characteristics and their WTP for the HSR.

## 2. Materials and Methods

### 2.1. Data Collection

This study used data previously collected as part of a larger multi-disciplinary project investigating consumer food purchasing and shopping behaviours. An online survey was administered to Australian participants via Qualtrics (Provo, UT, USA) between November 2014 and February 2015. A market research company (MyOpinions.com.au) was employed to disseminate the survey to registered panellists. All participants were required to be over 18 years of age and the primary grocery shopper for their household. Quotas were established for age, gender and location to generate a representative sample of Australian consumers. Adequate male representation was set at 30%, which was the estimated proportion of males principally responsible for grocery shopping in Australia [30]. Residing in one of five major Australian states (South Australia, New South Wales, Queensland, Victoria and Western Australia) was also an inclusion criterion for participation. The quotas set for age and location were reflective of the Australian general population [31] at the time of survey administration. Internet access was necessary for participation as the survey was disseminated online. No further inclusion/exclusion criteria were applied.

Survey items included for the exploration of consumer valuation of the HSR were: 12 socio-demographics questions; a food security indicator [32]; a modified version of a WTP scenario involving nutrition labelling [19]; and general questions related to perceived diet healthiness and label reading skills. The socio-demographic variables investigated included age, gender, income, education, occupation and family size. The short form (6-item) of the US Household Food Security Survey Module (HFSSM) [32] was utilised to categorise food security status. This food security indicator has been extensively validated and has comparable accuracy to the longer version with reduced respondent burden [33]. Food security status was classified into three levels: (1) High-Marginal, (2) Low and (3) Very Low, in accordance with the HFSSM user guide [32]. A more detailed methodology of the categorisation of the socio-demographic variables and food security status used in this study has been published previously [34].

### 2.2. Willingness to Pay

**Figure 1:**
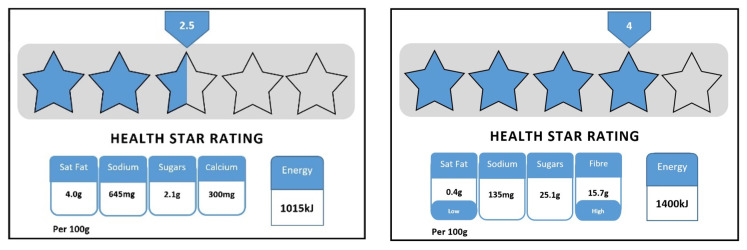
The graphic illustrates an example of the Health Star Rating front-of-pack label for a two-and-a-half-star food and a four-star food. (The Health Star Rating graphic included in the survey is not the official Health Star Rating (HSR) logo and was provided as an example at a time when the HSR was in development.)

To analyse and assess consumers’ WTP for a product displaying the HSR, we employed contingent valuation, a type of discrete choice experiment where consumers are asked to value the realisation of a scenario [26]. The valuation scenario presented in this study was adapted from a previous study that assessed consumers’ valuation of nutrition labels [19] and was described to participants as follows:

Suppose that a box of cookies also carries a government endorsed health star rating (from ½ star to 5 stars, with ½ star increments) similar to energy ratings that are featured on fridges and washing machines. The number of stars will be calculated using a government endorsed system with more stars for healthier food, such as those below: (see Figure 1).

**Figure 2:**
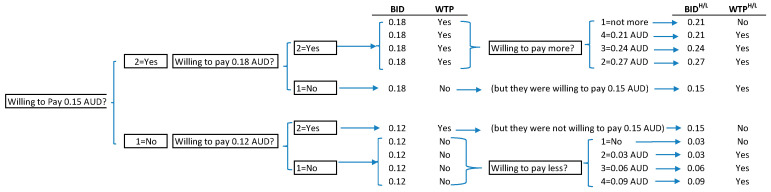
Questioning sequence and double-bounded dichotomous choice contingent valuation model setting. BID, bid (the hypothetical additional amount proposed for the Health Star Rating); WTP, willingness to pay; AUD, Australian dollars.

The induction modes for WTP responses in contingent valuation studies include bidding games, dichotomy, payment cards and open-ended questionnaires. Given the specific nature of this study and the related research instrument [19], a double-bounded dichotomous choice contingent valuation model (DCCVM) was selected. This modelling perspective is more information intensive and asymptotically more efficient than its single-bounded version [16]. Specifically, the double-bounded model requires participants to face a two-sequence bid offer, with the level of the second bid contingent upon the level of the first bid. A first bid (*Bid*) is offered to the participant and depending on the acceptance or not (*yes/no*), the second amount will be higher (*Bid^H^*) or lower (*Bid^L^*), respectively. This provides a censor or bound of the respondents’ WTP. The three survey questions utilised in the WTP scenario for this study are presented in Appendix A. In order to provide the required setting and be able to test the log-likelihood of the WTP underlying the econometric model, we based the DCCVM on the amounts presented in Question 2 as the first bid values (0.18 AUD, 6% of the price; or 0.12 AUD, 4% of the price) and then considered Question 3 or Question 1 amounts as second bid amounts. Figure 2 displays the DCCVM setting utilised for this study.

For a generic respondent *i*, we can denote the first bid amount by *BID_i_* and the second bid amount by *BID^H^_i_* if the respondent answers “yes” to the first offer or *BID^L^*_i_ if the respondent answers “no” to the first offer. Assuming a standard logistic function *F*, the probability that the answer is “yes” to both offers is given by:

(1) $$P^{yy}\left(BID_{i},BID_{i}^{H}\right)=1-F\left(BID_{i}^{H}\right)=\frac{1}{e^{\left(-\alpha +\beta BID_{i}^{H}+\gamma Z_{i}\right)}}=1+e^{\left(\alpha -\beta BID_{i}^{H}-\gamma Z_{i}\right)}$$

where *α*, *β* and *γ* are the parameters to be estimated through the maximisation of the log-likelihood of (1) and *Z* is a vector of the explanatory variables. Similarly, the probability that a respondent *i* answers “no” to both offers is equal to:

(2) $$P^{nn}\left(BID_{i},BID_{i}^{L}\right)=F\left(BID_{i}^{L}\right)=1-\frac{1}{e^{\left(-\alpha +\beta BID_{i}^{L}+\gamma Z_{i}\right)}}=1-e^{\left(\alpha -\beta BID_{i}^{U}-\gamma Z_{i}\right)}$$

The probability that respondent *i* answers “yes” to the first offer and “no” to the second, or “no” to the first offer and “yes” to the second is, respectively, defined by:

(3) $$P^{yn}\left(BID_{i},BID_{i}^{H}\right)=F\left(BID_{i}^{H}\right)-F\left(BID_{i}\right)=\left\{1-\frac{1}{e^{\left(-\alpha +\beta BID_{i}^{H}+\gamma Z_{i}\right)}}\right\}-\left\{1-\frac{1}{e^{\left(-\alpha +\beta BID_{i}^{}+\gamma Z_{i}\right)}}\right\}=\;e^{\left(\alpha -\beta BID_{i}^{}-\gamma Z_{i}\right)}-e^{\left(\alpha -\beta BID_{i}^{U}-\gamma Z_{i}\right)}$$

and

(4) $$P^{ny}\left(BID_{i},BID_{i}^{L}\right)=F\left(BID_{i}\right)-F\left(BID_{i}^{L}\right)=\left\{1-\frac{1}{e^{\left(-\alpha +\beta BID_{i}^{}+\gamma Z_{i}\right)}}\right\}-\left\{1-\frac{1}{e^{\left(-\alpha +\beta BID_{i}^{L}+\gamma Z_{i}\right)}}\right\}=\;e^{\left(\alpha -\beta BID_{i}^{L}-\gamma Z_{i}\right)}-e^{\left(\alpha -\beta BID_{i}^{}-\gamma Z_{i}\right)}$$

Consistent with the literature [19,35,36,37], we included socio-demographic characteristics as explanatory variables. Additionally, given the nature of this study, other explanatory variables linked to consumer perception of own diet healthiness, as well as food security status, were included. Therefore, the functional form of the WTP model including all the relevant variables in the study can be represented by the following equation:

(5) $$\begin{array}{cc} WTP_{i}=\alpha +\beta BID_{i} & +\overset{5}{\underset{n=1}{\sum }}\gamma_{1,n}Age_{n_{i}}+\gamma_{2}Gender_{i}+\gamma_{3}FamilySize_{i}\\ & +\;\overset{3}{\underset{n=1}{\sum }}\gamma_{4,n}Education_{n_{i}}+\overset{2}{\underset{n=1}{\sum }}\gamma_{5,n}Income_{n_{i}}+\overset{3}{\underset{n=1}{\sum }}\gamma_{6,n}HealthyDiet_{n_{i}}\\ & +\overset{4}{\underset{n=1}{\sum }}\gamma_{7,n}Hstar_{n_{i}}+\overset{2}{\underset{i=1}{\sum }}\gamma_{8,n}FoodSecurity_{n_{i}}+\varepsilon_{i} \end{array}$$

Table 1 defines all the variables included in the model. Maximisation of the related log-likelihood function was performed with R version 3.6.1 [38] using the DCchoice package [39], a package for analysing dichotomous choice contingent valuation data. Once the estimation of the parameters had been conducted, the mean and median WTP and related 95% confidence intervals were computed using different methodologies. The Krinsky–Robb [40] method, as well as bootstrapping, was applied to avoid specific limitations and increase the reliability of results [41] (p.246).

A supplementary analysis was conducted using a chi-square test to determine if a relationship existed between food security status and the perceived usefulness of the HSR when shopping.

### 2.3. Ethics

This study was conducted according to the guidelines laid down in the Declaration of Helsinki, and all procedures involving research study participants were approved by the Edith Cowan University’s Human Research Ethics Committee (# 11118). Written informed consent was obtained from all subjects prior to the start of the online survey.

## 3. Results

Of those consumers invited to participate, 1024 respondents completed the survey in its entirety (response rate: 18.8%). The majority of respondents were women (69%), aged 45–54 years (21%), and vocational education was the most frequently reported (37%) highest level of education. In addition, most respondents perceived their diet to be healthy (73%) and had High-Marginal food security (63%). Table 2 provides the consumers’ socio-demographic details and the parameter estimates for the DCCVM. The most significant factor for WTP was the bid amount. Consumers’ WTP significantly decreased as the bid amount (or price premium) presented in the scenario increased (*p* < 0.001). Grocery shoppers who agreed (*p* = 0.001), or strongly agreed (*p* < 0.001), with the statement ‘A HSR would make it easier for me to make selections when shopping’ were significantly more WTP for the HSR than those shoppers who strongly disagreed with this statement. Socio-demographic variables including gender, age, family size, education, income level and food security status were not significantly associated with consumers’ WTP for the HSR. One exception to this was the age group of 35–44 years, which had a slight negative association with WTP when compared to the most senior consumers surveyed (65–84 years) (*p* = 0.041). In addition, grocery shoppers who perceived their diet to be healthy (*p* = 0.004) or very healthy (*p* = 0.025) were more WTP for the HSR than shoppers who perceived their diet to be very unhealthy.

**Figure 3:**
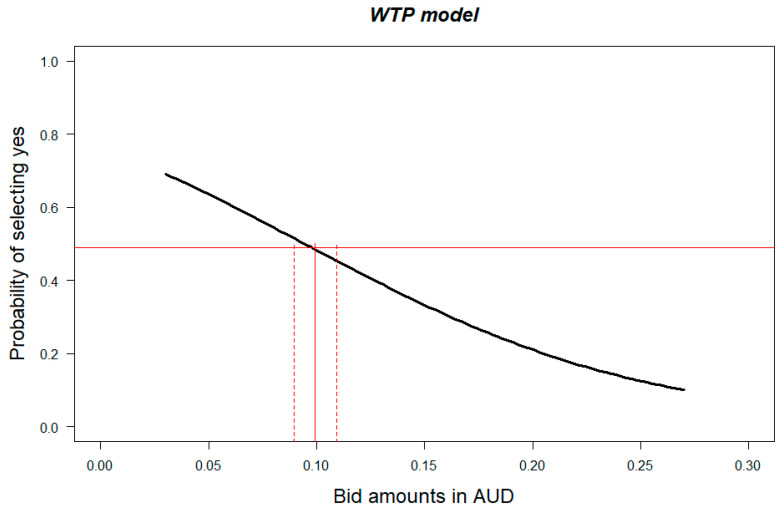
Price premium estimates in Australian dollars (AUD) for a box of cookies with the Health Star Rating on the front of the pack, where a box without the Health Star Rating costs AUD 3.00.

Figure 3 presents the resulting probability curve of a “yes” response to the hypothetical bid amounts related to the inclusion of the HSR on the food product. In Figure 3, the solid red horizontal line represents the 0.5 probability of a “yes” response, and the solid and dashed red vertical lines are the median WTP estimate and its 95% confidence interval, respectively.

Analysis showed that 40% (*n* = 409) of consumers were willing to pay the AUD 0.15 price premium for the HSR to be on the front of the food package, with 22% (*n* = 229) willing to pay AUD 0.18 or even more (10% AUD 0.21, 3% AUD 0.24 and 3% AUD 0.27). Of the 60% (*n* = 615) that were not willing to pay the initial AUD 0.15 price premium, 14% (*n* = 142) agreed to pay AUD 0.12 and 10% (*n* = 101) were willing to pay less (7% AUD 0.03, 2% AUD 0.06, 1% AUD 0.09). A total of 36% (*n* = 372) of consumers were not willing to pay additional money for the HSR to be on the front of the food package. The WTP mean, median and related 95% confidence intervals were calculated using both Krinsky and Robb [40] simulation and bootstrapping and are presented in Table 3. On average, the price premium that consumers were willing to pay for having an HSR on the front of the package was AUD 0.11 (or 3.67% of the initial product price without the HSR).

The supplementary analysis conducted using a chi-square test found a significant relationship (*p* = 0.008) between food security status and the perceived usefulness of the HSR when shopping. The results indicate that consumers classified as having Low food security, followed by the Very Low food security group, were the least likely to disagree with the statement that ‘a HSR would make it easier for me to make selections when shopping’.

## 4. Discussion

Our results indicate that almost two-thirds of Australian consumers were willing to pay more for a product with the HSR FoPL and on average agreed to pay up to 3.7% of the price of the product without the HSR. These results resemble the findings from a similar Spanish WTP study [19]. Loureiro et al. [19] examined value for nutrition labelling in general, rather than specifically FoPL, and found that Spanish consumers (*n* = 400) were willing to pay a price premium of 10.6% of the price of a box of cookies without a nutrition label.

The general lack of socio-demographic variables (age, income, education) having significant influence on WTP observed in this study, was also observed in the similar Spanish study [19]. However, the Spanish findings were only applicable to consumers who identified as not having any food-related health problems (i.e., health problems that require food intake control) [19]. Conversely, those individuals who identified as having food-related health problems were more willing to pay if they were older or had higher income or education levels.

In addition, our results indicate that consumer food security level was not associated with WTP for the HSR FoPL. Overall, this suggests that the most disadvantaged groups in our community (i.e., individuals with low food security, low income and low education) value the HSR similarly to other more advantaged groups. Interestingly, Gregori et al. [42] found that European consumers’ were more willing to pay when they have a low income or low education level, whereas they found high income consumers were less willing to pay for FoPL.

Not surprisingly, WTP was positively influenced by consumers who agreed that the HSR would make it easier to select products when shopping. Consumers who agreed with this perspective were more likely to pay a higher bid when compared with consumers who strongly disagreed. Furthermore, our results indicate that consumers who are considered food insecure are more likely to find the HSR more useful than their food secure counterparts. A potential explanation for the Low food security group identifying the HSR as most useful in our results could be that this group still have enough financial resources to have some ability to make choices when food shopping, while those consumers who have Very Low food security do not. Previous research has indicated that vulnerable populations are less likely to understand or use the detailed nutrition information panels (back of pack information) [43,44]. Interpretive FoPL could make food selection easier for vulnerable populations, but more research is warranted to clarify this relationship.

In regard to age, previous value elicitation studies suggest that older age groups (>45 years) are more willing to spend more money for nutrition labelling [42]. Our results somewhat agree with this trend, as the 35–44 year old grocery shoppers had significantly less value for the HSR than the more senior grocery shoppers (65–84 years). This trend may be reflective of people being more health conscious and consuming a healthier diet in the later years [45]. However, some researchers speculate that increasing age enhances WTP only when health problems are present, and older consumers are not likely to value nutrition labels more than younger consumers until they develop diet-related health problems [19]. Other studies [42] have shown that consumers who perceive themselves as obese are more likely to pay for FoPL, independent of their age. More research is required to determine the association between age, health status and WTP for nutrition labelling in the Australian population.

Consistent with the literature [42], we found suggestive evidence that increasing family size has a positive association with WTP. This trend may be reflective of consumers’ perceived lack of time for label reading and preference for the greater simplicity of interpretive FoPL when from a larger family. Alternatively, as others have described [46], this may be reflective of ‘nutritional altruism’, where food choice is influenced by consumers wanting to ensure the health of their family. In addition, consumers who perceived their diet to be healthy were willing to pay more compared to those who perceived their diet to be very unhealthy. It is well known that consumers are more likely to use nutrition labels when they have an interest in healthy eating, have a health goal in mind, or have better food and nutrition literacy [47,48]. This finding may suggest that consumers who put a high value on healthy eating also trust that the Government endorsed HSR is correctly identifying healthier food choices. Consumer trust and confidence in the HSR system has been reported to have grown in Australia and New Zealand over time [9]; in Australia, trust in the HSR had increased from 38% in 2015 to 61% in 2018 [49].

Encouragingly, our results suggest the HSR FoPL may have the capacity to evade the usual socio-economic determinants of health (lower income and education) that often inhibit the success of other public health nutrition initiatives.

### 4.1. Study Limitations

The findings of this study are limited to consumer WTP for the HSR on one food product. More research is needed to assess other food and beverage products, as WTP may vary according to the frequency in which products are purchased and the nutritional composition of products [19]. For example, research has shown that food type (cornflakes vs. cookies) influences WTP, where a larger difference in WTP has been observed for healthier relative to less healthy varieties of cornflakes compared to varieties of cookies [27]. Based on this evidence, it is possible that the average price premium that consumers were willing to pay in this study for the HSR on a box of cookies may have been higher if a food from a healthier food category was investigated.

The findings were based on self-reported data, which are subject to social desirability bias, and consumers were recruited through an online marketing company, which can be subject to sampling bias. Our study utilised a hypothetical method to determine WTP and therefore the results may be subjected to hypothetical bias. Hypothetical bias occurs in stated preference valuation studies when respondents report a WTP that is greater than what they actually pay using their own money in laboratory or field experiments [50]. Hypothetical methods tend to overestimate WTP when compared with consumers’ actual WTP [13], however, they provide an opportunity to obtain a larger sample size and reduce participant and researcher burden. This study was conducted six months after the HSR system was introduced to the Australian market and at this time there had been limited marketing and promotion of the new FoPL, with only a small number of products labelled with the system. Given that a high level of consumer trust and confidence in the HSR system now exists [9], it is feasible that consumer WTP for the HSR is higher than the estimates we have presented. However, this is the first study to investigate WTP in this context and as such it provides a valuable baseline for future research.

### 4.2. Policy Implications

Given that the majority of Australian consumers surveyed in this study valued and were willing to pay more for a product with the HSR, there appears to be an economic rationale for food manufacturers to display the HSR on their products. Yet, only 31% of Australian and 21% of New Zealand eligible products displayed the HSR as at June 2018 [9]. Public health advocates want the system to be mandated [49,51,52]. Mandating the system would ensure a more comprehensive and unbiased comparison of products because all eligible foods would be required to display health stars [52]. Other experts believe that the system should not be limited to only processed and packaged products but encourage a wider application of health stars across all foods to more accurately guide consumers in selecting a healthy diet [53]. Our evidence suggests that vulnerable populations may value the HSR and find it useful for the selection of healthier food options. However, there is a growing body of evidence indicating that disadvantaged people already struggle to afford to eat a nutritious diet [54,55]. Therefore, it is not recommended that products displaying the HSR have an additional cost added, as this may have an unintended consequence of reducing the affordability of healthier foods. Most importantly though, WTP for the HSR does not imply that consumers will eat a more nutritious diet. This can only be achieved when the mandated HSR system accurately represents the healthiness of foods and beverages. Experts advise that it would be counterproductive for the HSR system to be mandatory before current flaws in the nutrient profiling models that underpin the system have been resolved [52].

Two different methods are recommended to assess the accuracy of the nutrient profiling models that underpin FoPL: 1. Construct validity, the correlation between how the nutrient profiling model ranks the healthiness of foods in comparison to other measures; and 2. Criterion-related validity, the accuracy of the nutrient profiling model scores based on an externally derived objective measure of health [56,57]. The 2019 HSR Five Year Review [9] assessed the performance of the system using the following measures: percentage of eligible products displaying the HSR; accuracy of HSR compared to the HSR guidance materials; appropriateness of HSR compared to dietary guidelines; consumer awareness of the system; consumer trust in the system; influence on consumer purchasing; and product reformulation by food manufacturers. The HSR system has been reported to be performing well [9]; however, there are concerns as to how performance has been measured [58]. Two of the metrics used in the HSR review [9] reported to assess the accuracy of the system: the accuracy of HSR compared to the HSR guidance materials; and the appropriateness of HSR compared to dietary guidelines. The first of these metrics assessed accuracy by comparing the HSRs displayed on products with the HSR system guidance materials [9]. The guidance materials included the HSR style guide [59] and the HSR calculator [11]. Compliance with the guidance materials was found to be high, which indicated that manufacturers were using the system graphic as described, and correctly calculating the HSR score [9]. While it is important that industry correctly uses the guidance materials and the HSR calculator, it is imperative that the calculated HSR score provides an accurate representation of the healthiness of the product. The second metric used in the review to assess the accuracy of the HSR [9] somewhat addresses this issue by examining the alignment of HSRs displayed on products with dietary guidelines (construct validity). The World Health Organization recognises this approach as a simple method of validation and recommends that more complicated validation methods be conducted once the nutrient profiling model is developed [60]. One of the main limitations in assessing the healthiness of foods by considering their healthiness based on their dietary guidelines classification is the circularity in the argument [61,62]. This problem occurs because the construct of ‘healthiness’, as applied to foods measured by the nutrient profiling model and as applied to foods determined by the dietary guidelines, incorporates the same positive and negative nutrients/components. For example, saturated fat, sodium, total sugars, dietary fibre, protein, fruit and vegetables form the basis of the nutrient profiling models that underpin the HSR and are also the key nutrients/components in the dietary guidelines that contribute to a foods’ classification as a core or discretionary food. It is therefore unsurprising that the HSR Five Year Review [9] concluded that studies consistently showed the system to be well aligned with dietary guidelines. Researchers recommend that one way out of this circularity of reasoning is to make the constructs of ‘healthiness’ entirely independent of one another [61].

Indeed, three studies were conducted prior to the HSR Five Year Review [62,63,64] that assessed the construct validity of the HSR by comparing the system against the NOVA food classification system which is based on the extent of industrial food processing [65] rather than key nutrients/components. There is substantial evidence showing significant and graded associations between the dietary share of ultra-processed foods (as classified by NOVA) and dietary intakes high in sugar, saturated fats, and sodium and low in protein, fibre and potassium [66]. Furthermore, significant associations exist between the dietary share of ultra-processed foods and the incidence of obesity, cardiovascular and metabolic diseases, cancers, depression, gastrointestinal disorders, frailty in the elderly, and premature mortality [66]. These three validity studies [62,63,64] showed there was limited alignment between the HSR and the NOVA food classification system; however, the results of these studies were not included in the Five Year Review Report that assessed the performance of the HSR [9]. Since the publication of the 2019 HSR System Five Year Review Report, further research has identified misalignment between the HSR system and the NOVA food classification system. Researchers report the HSR is inadvertently providing a ‘health halo’, as 73% of the ultra-processed foods and beverages introduced into the Australian market during the five year HSR implementation period have displayed two and a half stars or more [67]. Level of food processing should be considered in the development of future iterations of the HSR [62], as demonstrated by other nutrient profiling models [68].

As described by the World Health Organization [60], an alternative way to avoid circularity of reasoning is to assess whether consuming healthy foods (as defined by the HSR) protects against adverse health outcomes (e.g., obesity, type 2 diabetes). This method assesses criterion-related validity and is recognised as the most robust way to assess the accuracy of nutrient profiling models; however, only a small proportion of models have been assessed using this method [56,69]. Importantly, there have been no validation assessments conducted to assess the ability of the HSR to predict health outcomes [49]. There is concern that the HSR algorithm has misrepresented nutrition science [58] and it is recommended that the system undergo further high level validation assessments [49] to confirm accuracy. FoPL that have undergone robust validity testing to demonstrate accuracy in measuring the healthiness of foods will be more efficient in improving the food environment for consumers than labels which are less extensively validated. The potential efficiencies of extensively validating the nutrient profiling models that underpin the HSR include: 1. improved health outcomes; 2. cost effectiveness; and 3. reduced environmental impacts.

Firstly, improved health outcomes are more likely following multiple robust validation assessments of the HSR system. Such assessments will ensure that products allocated five stars are unequivocally healthier than products with lower star ratings. As such, consumers’ WTP more for products with the HSR will be met with healthier food choices and a greater likelihood of positive health outcomes. A systematic review of the literature [69] that summarised the key characteristics of existing nutrient profiling models found that information on validity testing could not be identified for 58% of models. For those models for which validity testing could be identified, construct validity was the most commonly reported, and criterion validity testing was reported for only 10% of existing models. There is an urgent need for studies to assess the criterion-related validity of the nutrient profiling models that underpin the HSR system.

Secondly, cost effectiveness can only be achieved if the HSR system is accurate. The cost benefit analysis [12] that was conducted to assess the costs and the potential benefits from implementing the HSR can only be realised on the presumption that the HSR system is valid, that is, the nutrient profiling models that underpin the system accurately measure the healthiness of food and beverage items.

Thirdly, there are environmental consequences associated with the production of food which include: greenhouse gas emissions, use of natural resources, pressure on diversity, packaging and food waste [70]. Sustainable diets are diets with low environmental impacts and which provide present and future generations with food and nutrition security and a healthy life [71]. Validated models can add to the efficiency of sustainable diets by limiting the environmental consequences that are associated with food production and consumption to those foods which have a more positive health benefit [72]. For example, the reduced production and consumption of energy dense, ultra-processed packaged foods reduces the risk of diet-related chronic disease while optimising the use of environmental resources [70]. Currently, the HSR is frequently inadvertently contradicting Australian Dietary Guideline recommendations and promoting the consumption of discretionary and ultra-processed foods [58]. Policy makers need to consider the environmental and social impacts of consumers’ WTP for products displaying the HSR, as there is limited robust evidence to suggest the validity of the system or that consumption of higher star rated foods leads to better health outcomes. In addition, the majority of products that are eligible to display the HSR carry extensive packaging and often involve industrial processing.

## 5. Conclusions

This study demonstrates that Australian grocery shoppers are willing to pay more for a product that displays the HSR to indicate its relative healthiness. Our findings indicate that the HSR is valued by people of all socio-demographic backgrounds, including those individuals who are most disadvantaged. However, the potential benefit associated with consumers’ WTP more for the HSR is currently limited by the lack of guarantee of the systems’ accuracy. The potential efficiencies of thoroughly validating the nutrient profiling models that underpin the HSR to ensure accuracy include not only improved health outcomes for consumers, but also cost effectiveness and reduced environmental impacts.

## Figures and Tables

**Table 1:** Name, description and attributes of the study variables.

Variable	Description	Type	Levels/Range
WTP	Dependent variable accounting for the willingness to pay or not of the proposed bid amount	Binary	(Yes, No)
BID	Hypothetical additional amount proposed for the Health Star Rating	Numeric	(0.03 AUD~0.27 AUD)
Explanatory variables: Socio-demographic characteristics			
Age group	Age bracket of the respondent in years	Ordinal	(19–24, 25–34, 35–44,45–54, 55–64, 65–84)
Gender	The gender of the respondent	Binary	(Male, Female)
Family size	The sum of adults and children (<18 years) living in the household	Numeric	(1~9)
Education	Highest level of education completed.	Ordinal	(Secondary or less, Vocational, University, Others)
Income level	Level of income (i.e., wages/salaries, government benefits, pensions, allowances and other).	Ordinal	(Low, Mid, High)
Explanatory variables: Food and dietary factors			
Healthy diet	Level of perceived diet healthiness (How healthy would you say your diet was?)	Ordinal	(Very Unhealthy, Unhealthy, Healthy, Very Healthy)
Health Star Rating	Level of agreement with statement: ‘A Health Star Rating would make it easier for me to make selections when shopping’.	Ordinal	(Strongly Disagree, Disagree, Neither Agree or Disagree, Agree, Strongly Agree)
Food security	Level of food security	Ordinal	(High-Marginal food security, Low food security, Very Low food security)

WTP, willingness to pay; BID, bid (the hypothetical additional amount proposed for the Health Star Rating); AUD, Australian dollars.

**Table 2:** Parameter estimates of the willingness to pay equations.

Variable	Category	N (%) ^a^	Model Result		
			Estimate ± SE	z-Value	*p*-Value
BID (AUD)	Range 0.03–0.27	0.11 ± 0.08	−12.52 ± 0.49	−25.62	<0.001 ***
Gender	Male	320 (31%)	1.00 (Ref)		
	Female	704 (69%)	0.08 ± 0.13	0.64	0.521
Age	19–24	77 (8%)	−0.35 ± 0.28	−1.26	0.208
	25–34	189 (18%)	0.09 ± 0.22	0.42	0.674
	35–44	204 (20%)	−0.46 ± 0.22	−2.04	0.041 **
	45–54	215 (21%)	−0.12 ± 0.20	−0.62	0.538
	55–64	175 (17%)	0.01 ± 0.20	0.05	0.964
	65–84	164 (16%)	1.00 (Ref)		
Family size	Range 1–9	3.64 ± 1.32	0.10 ± 0.06	1.76	0.078
Education	Secondary or less	275 (27%)	1.00 (Ref)		
	Vocational	380 (37%)	0.09 ± 0.15	0.60	0.551
	University	360 (35%)	−0.04 ± 0.16	−0.27	0.790
	Other	9 (1%)	0.77 ± 0.62	1.24	0.213
Income level (AUD) ^b^	Low	322 (31%)	1.00 (Ref)		
	Middle	253 (25%)	0.22 ± 0.16	1.36	0.174
	High	244 (24%)	0.17 ± 0.16	1.03	0.305
Healthy diet ^c^	Very unhealthy	17 (2%)	1.00 (Ref)		
	Unhealthy	202 (20%)	1.88 ± 0.66	2.83	0.005 ***
	Healthy	746 (73%)	1.91 ± 0.66	2.91	0.004 ***
	Very healthy	59 (6%)	1.56 ± 0.70	2.24	0.025 **
Health Star Rating ^d^	Strongly disagree	30 (3%)	1.00 (Ref)		
	Disagree	132 (13%)	−0.09 ± 0.42	−0.22	0.828
	Neither agree or disagree	376 (37%)	0.63 ± 0.40	1.59	0.111
	Agree	356 (35%)	1.34 ± 0.40	3.38	0.001 ***
	Strongly agree	130 (13%)	1.68 ± 0.42	4.04	<0.001 ***
Food security ^e^	Very Low	171 (17%)	−0.11 ± 0.16	−0.65	0.518
	Low	204 (20%)	0.17 ± 0.15	1.15	0.248
	High-Marginal	649 (63%)	1.00 (Ref)		

Model Log-likelihood −1801.070; LR statistics 141.384 on 20 df, *p*-value <0.001; ** and *** represent the significance at 5% and 1%, respectively; **^a^** mean ± standard deviation is presented for numeric variables; SE = standard error; 1.00 (Ref) = reference level; BID, bid (the hypothetical additional amount proposed for the Health Star Rating); AUD = Australian dollars. ^b^ Income level refers to self-reported income for the household for 1 year including all wages/salaries, government benefits, pensions, allowances and other income. ^c^ Healthy diet refers to consumers’ perceived diet healthiness when asked: ‘How healthy would you say your diet was?’ ^d^ Health Star Rating refers to consumers’ agreement with statement: ‘A Health Star Rating would make it easier for me to make selections when shopping’. ^e^ Food security as categorised by the short form (6-item) of the US Household Food Security Survey Module [32].

**Table 3:** Consumers’ willingness to pay estimates and confidence intervals in Australian dollars (AUD) for a box of cookies with the Health Star Rating on the front of the pack, where a box without the Health Star Rating costs AUD 3.00.

	Estimate (AUD)	95% Confidence Interval [40]		95% Confidence Interval (Bootstrapping)	
		Lower Boundary (AUD)	Upper Boundary (AUD)	Lower Boundary (AUD)	Upper Boundary (AUD)
Mean	0.11 (3.67%)	0.10 (3.34%)	0.12 (4.00%)	0.10 (3.34%)	0.12 (4.00%)
Median	0.10 (3.34%)	0.09 (3.00%)	0.11 (3.67%)	0.09 (2.67%)	0.11 (3.67%)

## Appendix A

Q1: Since there are costs to the manufacturers to test and determine the star rating of each food product, would you be willing to pay more for having a health star rating at the front of the food package? Suppose the price of the box of cookies without the health star rating is $3, would you be willing to pay an additional 5% (or 15c) for the same product with the health star rating?			
Yes		No	
Q2: Given that you would pay an additional 5% (or 15c) for the same product with the health star rating, would you be willing to pay a little bit more, say 6% (or 18c)?		Q2: Given that you would not pay an additional 5% (or 15c) for the same product with the health star rating, would you be willing to pay a little bit less, say 4% (or 12c)?	
Yes	No	Yes	No
Q3: How much would you be willing to pay for the additional health star rating? 7% (or 21c) 8% (or 24c) 9% (or 27c) Not willing to pay more			Q3: How much extra would you be willing to pay? 3% (or 9c) 2% (or 6c) 1% (or 3c) Not willing to pay more
